# The K^+^ Channel Opener 1-EBIO Potentiates Residual Function of Mutant CFTR in Rectal Biopsies from Cystic Fibrosis Patients

**DOI:** 10.1371/journal.pone.0024445

**Published:** 2011-08-31

**Authors:** Eva K. Roth, Stephanie Hirtz, Julia Duerr, Daniel Wenning, Irmgard Eichler, Hans H. Seydewitz, Margarida D. Amaral, Marcus A. Mall

**Affiliations:** 1 Division of Pediatric Pulmonology and Allergy and Cystic Fibrosis Center, Department of Pediatrics III, University of Heidelberg, Heidelberg, Germany; 2 Translational Lung Research Center, University of Heidelberg, Heidelberg, Germany; 3 Pediatric Gastroenterology and Hepatology, Department of Pediatrics I, University of Heidelberg, Heidelberg, Germany; 4 Department of Pediatrics and Adolescent Medicine, University of Vienna, Vienna, Austria; 5 Department of Pediatrics and Adolescent Medicine, Albert Ludwigs University, Freiburg, Germany; 6 Centre of Human Genetics, National Institute of Health Dr. Ricardo Jorge, Lisboa, Portugal; 7 Department of Chemistry and Biochemistry, Faculty of Sciences, University of Lisboa, Lisboa, Portugal; Ludwig-Maximilians-Universität München, Germany

## Abstract

**Background:**

The identification of strategies to improve mutant CFTR function remains a key priority in the development of new treatments for cystic fibrosis (CF). Previous studies demonstrated that the K^+^ channel opener 1-ethyl-2-benzimidazolone (1-EBIO) potentiates CFTR-mediated Cl^−^ secretion in cultured cells and mouse colon. However, the effects of 1-EBIO on wild-type and mutant CFTR function in native human colonic tissues remain unknown.

**Methods:**

We studied the effects of 1-EBIO on CFTR-mediated Cl^−^ secretion in rectal biopsies from 47 CF patients carrying a wide spectrum of *CFTR* mutations and 57 age-matched controls. Rectal tissues were mounted in perfused micro-Ussing chambers and the effects of 1-EBIO were compared in control tissues, CF tissues expressing residual CFTR function and CF tissues with no detectable Cl^−^ secretion.

**Results:**

Studies in control tissues demonstrate that 1-EBIO activated CFTR-mediated Cl^−^ secretion in the absence of cAMP-mediated stimulation and potentiated cAMP-induced Cl^−^ secretion by 39.2±6.7% (*P*<0.001) via activation of basolateral Ca^2+^-activated and clotrimazole-sensitive KCNN4 K^+^ channels. In CF specimens, 1-EBIO potentiated cAMP-induced Cl^−^ secretion in tissues with residual CFTR function by 44.4±11.5% (*P*<0.001), but had no effect on tissues lacking CFTR-mediated Cl^−^conductance.

**Conclusions:**

We conclude that 1-EBIO potentiates Cl^−^secretion in native CF tissues expressing CFTR mutants with residual Cl^−^ channel function by activation of basolateral KCNN4 K^+^ channels that increase the driving force for luminal Cl^−^ exit. This mechanism may augment effects of CFTR correctors and potentiators that increase the number and/or activity of mutant CFTR channels at the cell surface and suggests KCNN4 as a therapeutic target for CF.

## Introduction

The early onset multiorgan disease cystic fibrosis (CF) is caused by more than 1,800 mutations in the *CFTR* gene and remains the most common fatal monogenetic disease in Caucasian populations [1], [2]. Therefore, the identification of strategies that improve impaired function of mutant CFTR remains a high priority in the development of causal therapies for patients with CF. The *CFTR* gene encodes a cAMP-regulated Cl^−^ channel that localizes to the luminal membrane of epithelial cells, where it plays an important role in transepithelial ion and fluid transport. *CFTR* mutations, via different molecular mechanisms, can reduce the number, or impair the regulation of CFTR Cl^−^ channels inserted into the apical cell membrane [3], [4], [5], [6], [7]. As a result, defective or reduced CFTR-mediated Cl^−^ secretion causes dehydration of epithelial surfaces and dysfunction of many epithelial organs including the small and large intestine, the pancreas, the hepato-biliary system, and the airways [2], [8], [9].

Current pharmacological strategies to rescue and/or improve CFTR function in CF focus on i) the development of CFTR correctors that increase the number of apical membrane CFTR Cl^−^ channels by improving the processing or synthesis of mutant CFTR; and ii) the development of potentiators that improve the open probability (P_O_) of mutant CFTR Cl^−^channels that are delivered to the cell surface, but exhibit impaired gating [3], [10]. In a recent phase 2 clinical trial in CF patients carrying the gating mutation G551D, the CFTR potentiator VX-770 [11] induced CFTR-mediated Cl^−^ secretion in nasal epithelia *in vivo* to levels of ∼20% of normal [12], [13]. A similar level of functional correction was obtained in CF patients with CFTR nonsense mutations after treatment with PTC124, a small molecule compound that improves read through at premature stop codons to produce full-length CFTR [14], [15]. Previous studies on the correlation between CFTR function and CF disease severity demonstrated that detection of residual CFTR function in this range (∼20% of normal) in native nasal or intestinal epithelia was associated with a less severe disease phenotype, but was not sufficient to prevent the onset and progression of CF organ disease [16], [17]. Therefore, additional pharmacological strategies that further improve Cl^−^ secretion through mutant CFTR channels may improve therapeutic effects of current CFTR potentiator and corrector compounds.

Besides activation of luminal CFTR Cl^−^ channels, CFTR-mediated Cl^−^ secretion relies on parallel activation of basolateral Ca^2+^- and cAMP-dependent K^+^ channels that hyperpolarize the membrane potential and generate the electrical driving force for luminal Cl^−^ exit [8], [18], [19]. A series of functional and genetic studies in cultured cells and mice demonstrated that the Ca^2+^-activated intermediate conductance K^+^ channel in the basolateral membrane of colonocytes is encoded by *KCNN4* and inhibited by clotrimazole [20], [21], [22], [23], [24], whereas the cAMP-dependent K^+^ channel is formed by the KCNQ1/KCNE3 complex and inhibited by the chromanol 293B [25], [26], [27], [28], [29], [30]. In this context, previous studies in colonic cells (T84) and mouse colon identified 1-ethyl-2-benzimidazolone (1-EBIO) as a compound that potentiates CFTR-mediated Cl^−^ secretion by coordinate activation of the basolateral Ca^2+^-activated K^+^ channels KCNN4 (SK4, IK1) and luminal CFTR Cl^−^ channels [31], [32], [33], [34], [35]. However, the effects of 1-EBIO on Cl^−^ secretion mediated by wild-type and mutant CFTR, and the role of KCNN4 and KCNQ1/KCNE3 K^+^ channels in native human colon have not been studied.

We previously demonstrated that bioelectric measurements in rectal biopsies mounted in perfused micro-Ussing chambers enable sensitive assessment of CFTR function in native human tissues [17], [36]. In this study, we used this approach to determine the effects of 1-EBIO on CFTR-mediated Cl^−^ secretion in rectal biopsies from non-CF individuals and CF patients carrying a wide spectrum of *CFTR* mutations. First, we used protocols to study the effect of 1-EBIO on the activity of luminal CFTR Cl^−^ channels. Second, we studied effects of 1-EBIO on basolateral Ca^2+^-activated and cAMP-dependent K^+^ channels and determined expression of KCNN4 and KCNQ1 K^+^ channel transcripts in human rectal biopsies. Finally, we determined the capacity of 1-EBIO to potentiate Cl^−^ secretion mediated by wild-type and mutant CFTR. The results of our studies provide novel insights into pharmacological modulation of mutant CFTR function in native human CF epithelia and identified co-activation of the basolateral K^+^ channel KCNN4 as a potential strategy to augment therapeutic effects of CFTR potentiator and corrector drugs.

## Materials and Methods

### Subjects

The study was approved by the Ethical Committees at the University Hospitals of Heidelberg, Freiburg, Lisbon and Vienna, and all subjects gave their written informed consent. For children under 18, parents obtained detailed information and gave their signed informed consent. The effects of 1-EBIO were studied in rectal biopsies from 47 CF patients (31 PI, 16 PS; mean age 10.4±1.4 yr, range 3 mo to 47 yr) and 57 age-matched non-CF controls (11.4±1.5 yr, range 6 mo to 45 yr) who were enrolled in the study between 1999–2011 at the CF Centers at the University Hospitals of Heidelberg, Freiburg, Lisbon and Vienna. The diagnosis of CF was established by clinical symptoms characteristic of CF, increased sweat Cl^−^ concentrations (≥ 60 mmol/L) and/or detection of disease-causing mutations in both *CFTR* alleles [37], [38]. Exocrine pancreatic insufficiency (PI) was defined by a history of malabsorption and fecal elastase E1 levels of <200 µg/g stool [39]. *CFTR* mutations were identified by sequential screening by allele specific PCR (Elucigene CF20 kit, Cellmark, Abingdon, UK) and denaturing gradient gel electrophoresis, followed by automated DNA sequencing of the 27 exons of the *CFTR* gene using the ABI Prism™ BigDye Terminator Cycle Sequencing Kit (Applied Biosystems, Weiterstadt, Germany) as previously described [17]. Genotypes of CF patients are summarized in table 1. In all control subjects, sweat Cl^−^ concentrations were normal (<30 mmol/L) and genetic screening for 20 common *CFTR* mutations was negative (Elucigene CF20 kit, Cellmark, Abingdon, UK).

**Table 1:** *CFTR* genotypes of CF patients with no detectable Cl^−^ secretion (CF_absent_) and residual Cl^−^ secretion (CF_residual_) in native rectal epithelia.

CF_absent_		CF_residual_	
*CFTR* genotype	Number of individuals	*CFTR* genotype	Number of individuals
F508del/F508del	10	F508del/Y161C	1
F508del/W57X	1	F508del/V232D	1
F508del/G85E	3	F508del/R334W	2
F508del/120del23	1	F508del/T338I	1
F508del/182delT	1	F508del/I1234V	1
F508del/G542X	1	F508del/3272-26 A>G	1
F508del/A561E	1	F508del/3849+10 kb C>T	1
F508del/Y1092X	1	F508del/4005+5727 A>G	1
F508del/N1303K	1	F508del/G576A	1
F508del/1525-1 G>A	2	N1303K/R334W	1
F508del/Q39X	1	F1052V/M1137R	1
F508del/Q552X	1	1898+3 A>G/1898+3 A>G	1
G85E/G85E	1	R334W/3199del6	1
Q552X/R1162X	1	R334W/X	1
A561E/A561E	2	dele2,3/X	1
R764X/1717-1 G>A	1		
R1158X/2183AA>G	1		
R1158X/R560T	1		

### Ussing Chamber Experiments

Superficial biopsies of the rectal mucosa (∼2 to 3 mm in diameter) were collected by endoscopic forceps biopsy, immediately stored in ice cold tissue medium (medium 199 containing Hank's salts, L-glutamine and 25 mmol/L HEPES complemented with 5 mmol/L glycine and 0.5 mmol/L Sodium-DL-β-hydroxybutyrate) and mounted in perfused micro-Ussing chambers (open area ∼0.95 mm^2^) as previously described [19], [40]. In brief, the luminal and basolateral surfaces of the epithelium were perfused continuously with a solution of the following composition (mmol/L): NaCl 145, KH_2_PO_4_ 0.4, K_2_HPO_4_ 1.6, D-glucose 5, MgCl_2_ 1, Ca-gluconate 1.3, pH 7.4, at 37°C. Experiments were performed under open-circuit conditions. Values for the transepithelial voltage (V_te_) were referenced to the serosal surface of the epithelium. Transepithelial resistance (R_te_) was determined by applying intermittent (1 s) current pulses (ΔI = 0.5 µA). The equivalent short circuit current (I_sc_') was calculated according to Ohm's law from V_te_ and R_te_ (I_sc_' = V_te_/R_te_) after appropriate correction for fluid resistance [19], [40].

### Experimental Protocols and Analysis of Ussing Chamber Experiments

Rectal tissues were equilibrated for 40 min in the presence of amiloride (10 µmol/L, luminal) to block electrogenic Na^+^ absorption and indomethacin (10 µmol/L, basolateral) to inhibit prostaglandin E2 synthesis and endogenous cAMP formation. Previous studies showed that under these experimental conditions, CFTR activity is inhibited and Ca^2+^-dependent activation with carbachol (CCH, 100 µmol/L, basolateral) results in a transient lumen-positive I_sc_' response reflecting K^+^ secretion that is unmasked in the absence of anion secretion [8], [19], [40]. When CFTR Cl^−^ channels are activated under these conditions, Ca^2+^-dependent co-stimulation produces lumen-negative (Cl^−^ secretory) responses by activation of basolateral Ca^2+^-activated K^+^ channels that increase the electrical driving force for luminal Cl^−^ exit [19], [40], [41]. To determine the effects of 1-EBIO on CFTR-mediated Cl^−^ secretion in the absence of cAMP-mediated activation (indomethacin), we therefore measured the effects of 1-EBIO (500 µmol/L, basolateral) on basal I_sc_' and CCH-induced I_sc_', and determined the effects of the CFTR inhibitor CFTR_inh_-172 (20 µmol/L, basolateral) on 1-EBIO-induced responses.

To study the effect of 1-EBIO on basolateral Ca^2+^-activated and cAMP-dependent K^+^ channels that generate the driving force for CFTR-mediated Cl^−^ secretion, control rectal tissues were stimulated with 3-isobutyl-1-methylxanthine (IBMX; 100 µmol/L, basolateral) and forskolin (1 µmol/L, basolateral) to obtain maximal cAMP-mediated activation of CFTR, and the effects of 1-EBIO on cAMP-induced I_sc_' and CCH-induced I_sc_' were measured before and after adding either clotrimazole (30 µmol/L, basolateral), an inhibitor of the basolateral Ca^2+^-activated K^+^ channel KCNN4 [20], [22], [23], or 293B (10 µmol/L, basolateral), an inhibitor of the cAMP-dependent heteromeric KCNQ1/KCNE3 K^+^ channel [25], [26], [28]. Dose-response curves for the effects of 1-EBIO in the presence of cAMP-mediated activation (IBMX/forskolin) were obtained by measuring the change in I_sc_' induced by exposing tissues to increasing concentrations of 1-EBIO (10^−8^ to 10^−3^ mol/L) and plotted as 1-EBIO-induced I_sc_' normalized to the maximal 1-EBIO-induced I_sc_' (I_sc_' max), and EC_50_ values were determined by fitting dose-response data to the Hill equation using Origin version 6.1 (OriginLab Corp., Northampton, MA).

To determine potentiator effects of 1-EBIO on CFTR-mediated Cl^−^ secretion in native control and CF rectal epithelia, tissues were activated with IBMX and forskolin and effects of 1-EBIO on cAMP-induced I_sc_' and CCH-induced I_sc_' were measured in control tissues, CF rectal tissues with no detectable Cl^−^ secretion (CF_absent_) and CF tissues with residual cAMP-mediated Cl^−^ secretion (CF_residual_). In the presence of cAMP stimulation, CCH induced i) a transient monophasic lumen-negative (Cl^−^ secretory) response (control tissues), ii) a transient monophasic lumen-positive (K^+^ secretory) response (CF_absent_ tissues), or iii) a biphasic response characterized by a lumen-positive peak followed by a lumen-negative deflection (CF_residual_) [17], and effects of 1-EBIO on CCH-induced Cl^−^ secretion were determined from lumen-negative deflections (control and CF_residual_ tissues) or the lumen-positive plateau (CF_absent_ tissues). In a subset of experiments, we used CFTR_inh_-172 (20 µmol/L, luminal), and bumetanide (100 µmol/L, basolateral), an inhibitor of the Na^+^-K^+^-2Cl^−^ cotransporter (NKCC1), to block transepithelial Cl^−^ secretion [40]. For different experimental protocols, bioelectric measurements were generally performed in one rectal biopsy per individual. Occasionally, more than one sample from one individual was studied by the same experimental protocol, and data were averaged to obtain a single value for each individual subject.

### Reverse Transcriptase Polymerase Chain Reaction

Rectal biopsies were stored in RNAlater (Ambion, Darmstadt, Germany) and total RNA was isolated using the RNeasy Mini Kit (Quiagen, Hilden, Germany) and reverse transcribed into cDNA using Superscript III (Invitrogen, Karlsruhe, Germany). Sequences specific for KCNN4 (405 bp) and KCNQ1 (738 bp) were amplified by PCR (94°C for 1 min, 60–62°C for 1 min, 72°C for 2 min, 35 cycles) using the following custom made primers (Eurofins MWG Operon, Ebersberg, Germany): KCNN4, 5′-GATTTAGGGGCGCCGCTGAC–3′ (sense) and 5′-CTTGCCCCACATGGTGCCC–3′ (antisense); KCNQ1, 5′-TTCTGGATGGAGATCGTG-3′ (sense) and 5′-GCCTTCCGGATGTAGATC–3′ (antisense), as previously described [42], [43]. PCR products were visualized by 0.9% (w/v) agarose gel electrophoresis using a 100 bp DNA ladder as a DNA size marker, and sequences were verified by sequencing (Seqlab, Göttingen, Germany).

### Chemicals and Compounds

Amiloride, indomethacin, carbachol, IBMX, forskolin, bumetanide and clotrimazole were all obtained from Sigma (Deisenhofen, Germany). 1-EBIO was obtained from Tocris bioscience (Avonmouth, UK), CFTR_inh_-172 from Calbiochem (Darmstadt, Germany) and 293B from Hoechst (Frankfurt, Germany). All chemicals used were of highest grade of purity available.

### Statistical Analysis

Data were analyzed with SigmaStat version 3.1 (Systat Software, Erkrath, Germany) and are reported as mean ± SEM (*n* =  number of individuals studied). Statistical analyses were performed using paired Student's t test and Wilcoxon signed rank test as appropriate, and *P*<0.05 was accepted to indicate statistical significance.

## Results

### 1-EBIO activates CFTR-dependent Cl^-^ secretion in native rectal epithelia

**Figure 1:**
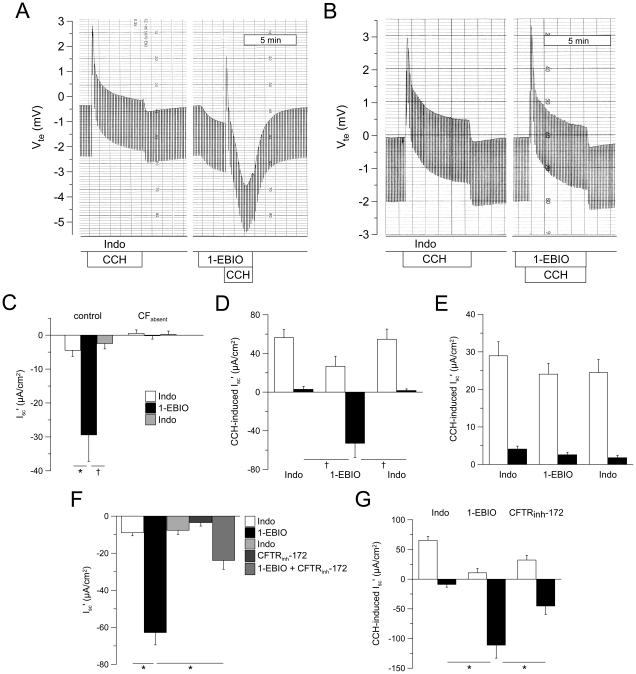
1-EBIO activates CFTR-mediated basal and cholinergic Cl^−^ secretion in human rectal biopsies. (A,B) Original recordings of effects of 1-EBIO (500 µM, basolateral) on basal and carbachol-induced (CCH) transepithelial voltage (V_te_) and transepithelial resistance (R_te_) across rectal biopsies from a control subject (A) and a CF patient carrying two severe *CFTR* mutations (R1158X/2183AA>G). (B) Experiments were performed in the presence of amiloride and indomethacin. Lumen-positive V_te_ responses reflect K^+^ secretion and lumen-negative responses reflect Cl^−^ secretion. R_te_ was determined from V_te_ downward deflections obtained by pulsed current injection. (C) Summary of effects of 1-EBIO on basal equivalent short-circuit current (I_sc_') in rectal biopsies from control subjects and CF patients with no detectable Cl^−^ secretion (CF_absent_). (D,E) Effects of 1-EBIO on CCH-induced peak (open bars) and plateau (closed bars) I_sc_' responses in control (D) and CF_absent_ rectal tissues (E). (F,G) Effect of CFTR_inh_-172 on 1-EBIO-induced Cl^−^ secretion (lumen-negative I_sc_') under basal conditions (F) and on carbachol-induced (CCH) Cl^−^ secretion in the presence of 1-EBIO (G) in rectal biopsies from control subjects. Data are presented as mean±SEM. n = 7–13 individuals per group. * *P*<0.001 and ^†^ *P*<0.01.

To study the effects of 1-EBIO on luminal CFTR Cl^−^ channels in native human rectal epithelia, tissues were pretreated with indomethacin to block endogenous cAMP-mediated CFTR activation. Under these conditions, V_te_ and I_sc_' approached ∼zero and cholinergic activation with CCH induced a monophasic lumen-positive response reflecting luminal K^+^ secretion (Fig. 1 A,C,D) indicating that luminal CFTR Cl^−^ channels were inactive [19], [40]. In control tissues, addition of 1-EBIO resulted in a sustained lumen-negative Cl^−^ secretory response (ΔI_sc_'  = −25.0±6.6 µA/cm^2^, n = 12, *P* = 0.001) (Fig. 1 A,C). In the presence of 1-EBIO, CCH induced a biphasic response with the lumen-positive K^+^ secretory peak followed by a lumen-negative peak reflecting a transient increase in 1-EBIO-induced Cl^−^ secretion (Fig. 1 A,D) [41]. Effects of 1-EBIO on basal and CCH-induced I_sc_' were completely reversible upon washout (Fig. 1 C,D). In rectal tissues from CF patients with two severe *CFTR* mutations and no detectable cAMP-mediated Cl^−^ secretion (CF_absent_) (Table 1), 1-EBIO failed to induce Cl^−^ secretion (ΔI_sc_' = −0.7±0.8 µA/cm^2^, n = 7, *P* = 0.3) and had no effect on CCH-induced I_sc_' (Fig. 1 B,C,E). In rectal biopsies from the control group, 1-EBIO-induced Cl^−^ secretion was significantly inhibited when tissues were pretreated with the CFTR inhibitor CFTR_inh_-172 (Fig. 1 F). Similar, CCH-induced Cl^−^ secretory (lumen-negative) responses detected in the presence of 1-EBIO were inhibited by CFTR_inh_-172 (Fig. 1 G). Taken together, these data provide pharmacologic and genetic evidence that 1-EBIO activated luminal CFTR Cl^−^ channel activity and transepithelial Cl^−^ secretion in native human rectal epithelia in the absence of cAMP-mediated stimulation.

### 1-EBIO activates basolateral Ca^2+^-dependent but not cAMP-dependent K^+^ channels in native rectal epithelia

**Figure 2:**
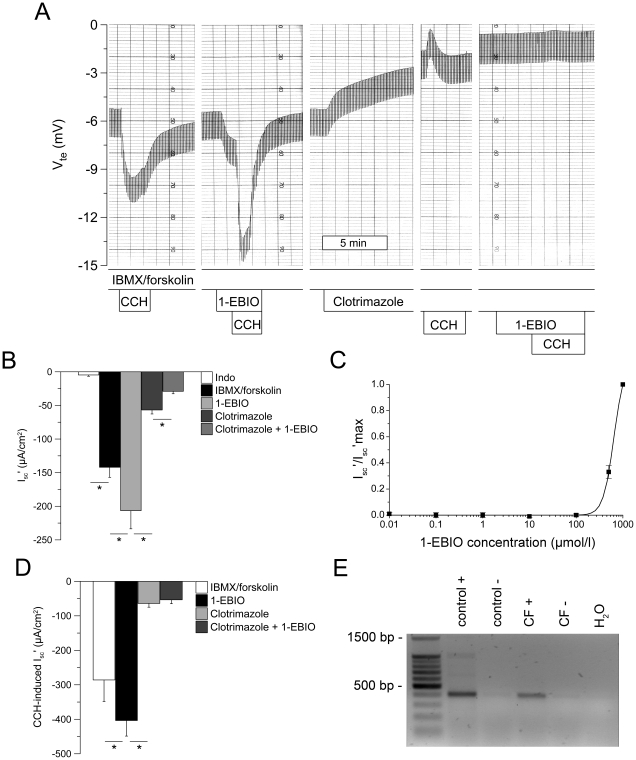
1-EBIO potentiates cAMP-mediated and cholinergic Cl secretion in human rectal biopsies and this effect is abrogated by inhibition of Ca^2+^-dependent K^+^ channels with clotrimazole. (A) Original recording of effects of 1-EBIO (500 µM, basolateral) on cAMP-induced Cl^−^ secretion (IBMX/forskolin) and cholinergic co-activation (CCH), and effects of clotrimazole (30 µM, basolateral) on Cl^−^ secretory responses in a rectal biopsy from a control subject. Experiments were performed in the presence of amiloride, indomethacin and IBMX/forskolin. (B) Summary of effects of 1-EBIO on cAMP-induced Cl^−^ secretion and inhibition by clotrimazole in rectal tissues from control subjects. (C) Concentration-response curve for 1-EBIO-induced Cl^−^ secretion was determined in the presence of cAMP-mediated activation (IBMX/forskolin). (D) Effects of 1-EBIO on CCH-induced Cl^−^ secretion in the presence of IBMX/forskolin and inhibition by clotrimazole in control rectal tissues. Data are presented as mean±SEM. n = 17 individuals per group. *P<0.001. (E) RT-PCR analysis revealed transcripts of the clotrimazole-sensitive Ca^2+^-activated K^+^ channel KCNN4 in rectal biopsies from control and CF subjects. The 405 bp KCNN4 fragment was only identified in the presence (+), but not in the absence of reverse transcriptase (-).

**Figure 3:**
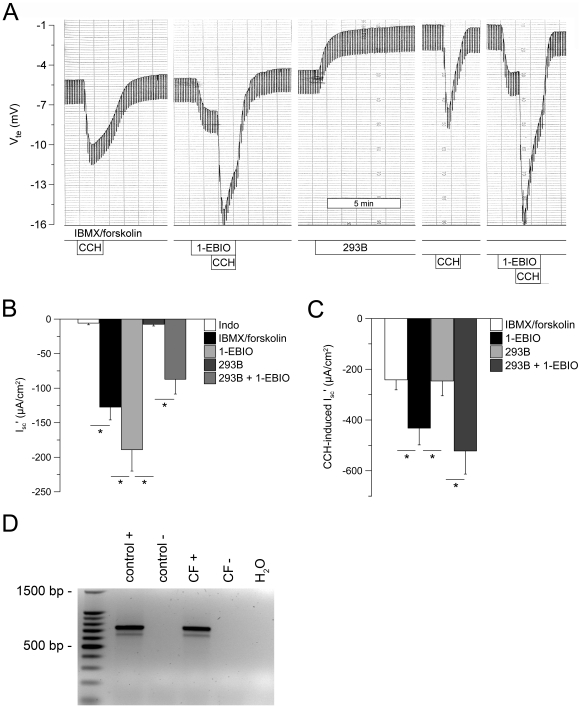
1-EBIO mediated augmentation of cAMP-induced and cholinergic Cl^−^ secretion in human rectal biopsies does not depend on 293B-sensitive cAMP-dependent K^+^ channels. (A) Original recording of effects of 1-EBIO (500 µM, basolateral) on cAMP-induced Cl^−^ secretion (IBMX/forskolin) and cholinergic co-activation (CCH), and effects of 293B (10 µM, basolateral) on Cl^−^ secretory responses in a rectal biopsy from a control subject. Experiments were performed in the presence of amiloride, indomethacin and IBMX/forskolin. (B, C) Summary of effects of 1-EBIO on cAMP-induced (B) and CCH-induced Cl^−^ secretion (C) in the absence and presence of 293B in rectal tissues from control subjects. Data are presented as mean±SEM. n = 19 individuals per group. *P<0.001. (D) RT-PCR analysis detected transcripts of the 293B-sensitive K^+^ channel KCNQ1 (728 bp fragment) in the presence (+), but not in the absence of reverse transcriptase (-), in rectal biopsies from control and CF subjects.

Next, we determined the effect of 1-EBIO on basolateral K^+^ channels in human rectal tissues. In these experiments, CFTR was first activated by maximal cAMP stimulation (IBMX/forskolin) to generate conditions under which basolateral K^+^ channels became limiting for CFTR-mediated Cl^−^ secretion [8], [30]. Under these conditions, we then tested the effects of clotrimazole, an inhibitor of the Ca^2+^-activated KCNN4 K^+^ channel (Fig. 2), and 293B, an inhibitor of the cAMP-dependent KCNQ1/KCNE3 K^+^ channel complex (Fig. 3), on 1-EBIO-induced responses in control rectal tissues.

Stimulation of tissues with IBMX and forskolin produced a large and sustained Cl^−^ secretory response that was significantly increased by 1-EBIO (ΔI_sc_' = −64.6±13.5 µA/cm^2^, n = 17, *P* = 0.001) in a reversible and dose-dependent fashion (EC_50_ = 583.8±21.2 µmol/l; n = 6) (Fig. 2 A,B,C). As expected from previous studies, cholinergic co-activation induced a transient increase in Cl^−^ secretion reflecting an increase in the driving force for CFTR-mediated Cl^−^ secretion generated by activation of basolateral K^+^ channels [8], [19]. CCH-induced Cl^−^ secretory responses were significantly increased after tissues were pretreated with 1-EBIO (Fig. 2 A,D). The effects of 1-EBIO on cAMP-induced (IBMX/forskolin) and CCH-induced Cl^−^ secretion were completely inhibited when tissues were perfused with clotrimazole (Fig. 2 A,B,D). RT-PCR analyses demonstrated that transcripts encoding for the clotrimazole-sensitive Ca^2+^-activated K^+^ channel KCNN4, which is pharmacologically activated by 1-EBIO and responsible for driving Ca^2+^-activated Cl^−^ in mouse colon [22], [23], [31], are expressed in native rectal tissues from control subjects and CF patients (Fig. 2 E).

Perfusion of rectal tissues with 293B abolished cAMP-induced Cl^−^ secretion as expected [19], and RT-PCR demonstrated expression of KCNQ1 transcripts in native rectal epithelia (Fig. 3A,B,D). However, in contrast to clotrimazole (Fig. 2), 293B did neither inhibit 1-EBIO-induced Cl^−^ secretion, nor the augmentation of cholinergic Cl^−^ secretion by 1-EBIO in the presence of cAMP activation (Fig. 3A,B,C). Collectively, these results indicated that 1-EBIO augments CFTR-mediated Cl^−^ secretion in human colon in the presence of cAMP stimulation by activation of the basolateral Ca^2+^-activated KCNN4 K^+^ channel, but not the cAMP-dependent KCNQ1/KCNE3 K^+^ channel complex, and suggested that 1-EBIO may potentiate residual CFTR-mediated Cl^−^ secretion in CF tissues via this mechanism.

### 1-EBIO potentiates residual CFTR-mediated Cl^−^ secretion in CF rectal biopsies

To test if pharmacological co-activation of the Ca^2+^-regulated KCNN4 K^+^ channel promotes CFTR-mediated Cl^−^ secretion in native CF tissues, we next determined the effects of 1-EBIO in rectal biopsies from CF patients carrying a large spectrum of *CFTR* mutations (Table 1). Following the equilibration in the presence of amiloride and indomethacin, CF tissues were stimulated with IBMX and forskolin to achieve maximal activation of luminal CFTR Cl^−^ channels and basolateral KCNQ1/KCNE3 K^+^ channels, and co-stimulated with CCH to activate Ca^2+^-regulated KCNN4 K^+^ channels, thus maximizing the driving force for CFTR-mediated Cl^-^ secretion [8], [19]. Based on Cl^−^ secretory responses observed with this protocol, tissues were stratified into two groups, namely one group including tissues expressing residual CFTR Cl^−^ channel function (CF_residual_) and another group with no detectable Cl^−^ secretion (CF_absent_) [17]. The CF genotypes of patients assigned to each group are summarized in table 1. Tissues from age-matched control subjects were included in these studies as a reference of wild-type CFTR function.

**Figure 4:**
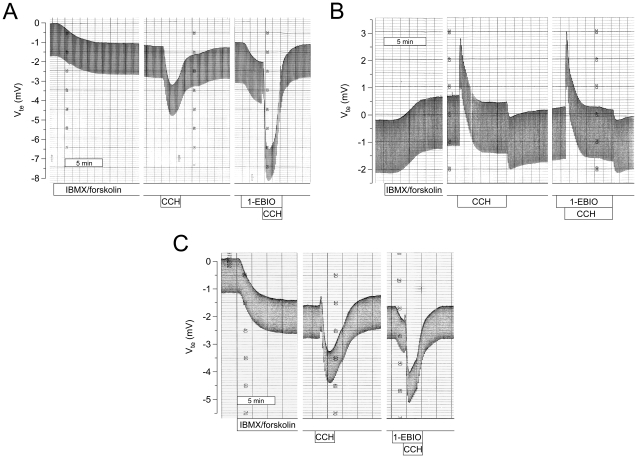
1-EBIO potentiates residual CFTR-mediated Cl^−^ secretion in CF rectal biopsies. (A–C) Original recordings of effects of cAMP-mediated (IBMX/forskolin) and cholinergic (CCH) activation, and effects of 1-EBIO (500 µM, basolateral) on transepithelial voltage (V_te_) and resistance (R_te_) in rectal tissues from a control subject (A), a CF patient with no detectable Cl^−^ secretion (CF_absent_; R1158X/2183AA>G) (B), and a CF patient with residual Cl^−^ secretion (CF_residual_; F508del/Y161C), as evidence by lumen-negative V_te_ responses (C). Experiments were performed in presence of amiloride and indomethacin. 1-EBIO potentiated cAMP-mediated and cholinergic Cl^−^ secretion in control and CF_residual_ rectal tissues, but did not induce Cl^−^ secretion in the CF_absent_ tissue.

**Figure 5:**
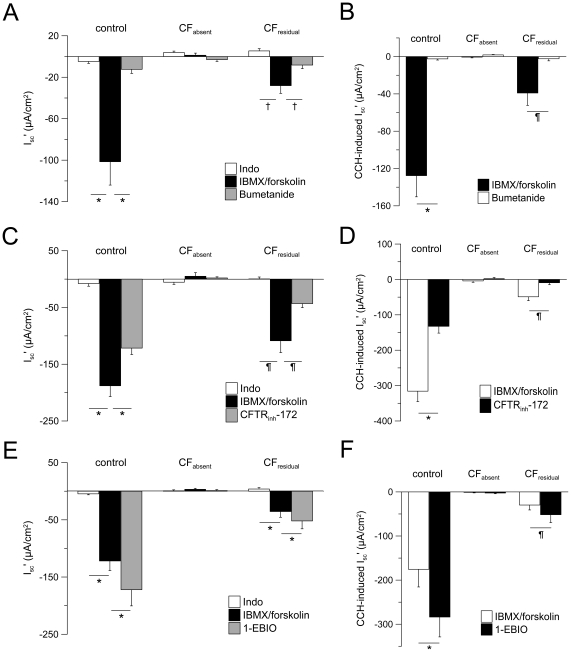
1-EBIO potentiates residual CFTR-mediated Cl^−^ secretion in CF rectal biopsies. (A–F) Summary of effects of bumetanide (100 µM, basolateral) (A,B), CFTR_inh_-172 (20 µM, basolateral) (C,D) and 1-EBIO (500 µM, basolateral) (E,F) on cAMP-mediated (IBMX/forskolin) and cholinergic (CCH) activation of equivalent short circuit current (I_sc_') in rectal biopsies from control subjects, CF patients with no detectable Cl^−^ secretion (CF_absent_) and CF patients with residual Cl^−^ secretion (CF_residual_). All experiments were performed in the presence of amiloride and indomethacin. Only lumen-negative peak responses or plateau responses are shown for cholinergic (CCH) activation. Data are presented as mean±SEM. n = 4–26 individuals per group. *P<0.001, ^†^ *P*<0.01 and ^¶^ *P*<0.05.

In the control group, cAMP-mediated stimulation induced a large Cl^−^ secretory response that was significantly increased by cholinergic co-activation as expected [19] (Fig. 4 and 5). In the CF_absent_ group, cAMP-mediated stimulation induced small lumen-positive responses and co-activation with CCH produced a monophasic lumen-positive I_sc_' response reflecting K^+^ secretion [40] (Fig. 4 and 5). In the CF_residual_ group, cAMP-mediated activation induced an attenuated but sustained lumen-negative Cl^−^ secretory response (Fig. 4 and 5). Consistent with previous studies [17], co-activation of CF_residual_ tissues with CCH produced biphasic responses with an initial lumen-positive (K^+^ secretory) deflection followed by a transient lumen-negative (Cl^−^ secretory) response (Fig. 4 and 5). Mean Cl^−^ secretory responses in CF_residual_ tissues accounted for ∼34% of cAMP-mediated Cl^−^ secretion and ∼17% of CCH-induced Cl^−^ secretion observed in control rectal tissues (Fig. 5E,F). Similar to control tissues, Cl^−^ secretory responses in CF_residual_ tissues were inhibited by bumetanide and CFTR_inh_-172 demonstrating that residual cAMP- and CCH-induced Cl^−^ secretion was mediated by CFTR (Fig. 5A–D).

Addition of 1-EBIO significantly increased cAMP-mediated Cl^−^ secretion in control tissues (ΔI_sc_' = −50.2±13.1 µA/cm^2^, n = 18, *P* = 0.001) and CF_residual_ tissues (ΔI_sc_' = −16.4±5.1 µA/cm^2^, n = 12, *P* = 0.001), but had no effect on CF_absent_ tissues (Fig. 4 and 5E). Of note, the percent increase in Cl^−^ secretion induced by 1-EBIO was similar in the control group (39.2±6.7%, n = 18, *P*<0.001) and CF_residual_ group (44.4±11.5%, n = 12, *P*<0.001). Further, 1-EBIO augmented CCH-induced Cl^−^ secretory responses by 85.1±12.2%, n = 18, (*P*<0.001) in control tissues and 72.8±21.4, n = 7 (*P*<0.05) in CF_residual_ tissues, but failed to induce Cl^−^ secretion in CF_absent_ tissues (Fig. 4 and 5F). Taken together, these data demonstrate that activation of basolateral Ca^2+^-activated KCNN4 K^+^ channels by 1-EBIO potentiates CFTR-mediated Cl^−^ secretion induced by cAMP-mediated and cholinergic activation in native CF rectal tissues that express mutant CFTR proteins with residual Cl^−^ channel function.

## Discussion

This study provides important information on the pharmacological modulation of CFTR-mediated Cl^−^ secretion in native human normal and CF rectal epithelia. First, by using experimental conditions under which CFTR Cl^−^ channels were inactive, we demonstrate that the benzimidazolone compound 1-EBIO [31], [32], [33], [34], [35] activated CFTR-mediated Cl^−^ secretion in normal rectal tissues in the absence of cAMP-mediated activation (Fig. 1). As previously shown for the CFTR opener genistein [41], 1-EBIO-induced Cl^−^ secretory responses were augmented by cholinergic stimulation, consistent with parallel activation of basolateral Ca^2+^-activated K^+^ channels that increase the driving force for CFTR-mediated Cl^−^ secretion [8], [19]. The notion that 1-EBIO acted as a CFTR Cl^−^ channel opener in the absence of cAMP-mediated activation was supported by the observations i) that 1-EBIO-induced Cl^−^ secretory responses were inhibited by the CFTR inhibitor CFTR_inh_-172, and ii) that 1-EBIO failed to induce Cl^−^ secretion in rectal epithelia from CF patients carrying two severe *CFTR* mutations that caused a lack of functional CFTR Cl^−^ channels in the apical cell membrane (Table 1) [7], [17]. Second, our results show that 1-EBIO activated coltrimazole-sensitive basolateral K^+^ channels in rectal epithelia (Fig. 2,3). This effect of 1-EBIO augmented CFTR-mediated Cl^−^ secretion in normal tissues when luminal CFTR Cl^-^ channels were fully activated, as evidenced by a substantial increase in steady-state and CCH-induced Cl^−^ secretory responses in the presence of cAMP stimulation. As shown in figure 2, this augmentation of Cl^−^ secretion by 1-EBIO was completely abrogated when tissues were pretreated with clotrimazole. In contrast, pretreatment of tissues with 293B had no effect on 1-EBIO-induced Cl^−^ secretion (Fig. 3). This pharmacological profile, together with mRNA transcripts analyses showing that both clotrimazole-sensitive KCNN4 K^+^ channels, as well as 293B-sensitive KCNQ1 K^+^ channels were expressed in rectal tissues, support the concept that 1-EBIO activates basolateral KCNN4 K^+^ channels and thereby increases the electrical driving force for CFTR-mediated Cl^−^ secretion in native human colon. The observation that 1-EBIO promoted transepithelial Cl^−^ secretion even in the absence of Ca^2+^-dependent stimulation (Fig. 2A–C) is consistent with previous patch-clamp studies in cultured colonocytes (T84) and heterologous cells, which demonstrated that 1-EBIO activates KCNN4 K^+^ channels at low levels of free Ca^2+^ [31], [44]. These results suggest that pharmacological activation of basolateral KCNN4 K^+^ channels can potentiate CFTR-mediated Cl^−^ secretion in the colon under basal conditions, as well as in the presence of secretagogues activating cAMP- and Ca^2+^ signaling pathways *in vivo*. Taken together, our data showing a coordinate activation of luminal CFTR Cl^−^ channels and basolateral KCNN4 K^+^ channels by 1-EBIO in human rectal epithelia are consistent with previous results in colonic cells [22], [31], [32] and mouse colon [23], [33], [34], [35]. These results thus confirmed that this dual mode of action of 1-EBIO is also operative in native human colon, and formed the rationale for the hypothesis that 1-EBIO may potentiate Cl^−^ secretion in native CF tissues expressing CFTR mutants with residual Cl^−^ channel function.

We tested this hypothesis by studying the effects of 1-EBIO in rectal tissues from CF patients carrying a wide spectrum of CF genotypes (Table 1). These measurements focused on potentiator effects of 1-EBIO in cAMP pre-stimulated tissues and demonstrated that pharmacological activation of basolateral KCNN4 K^+^ channels by 1-EBIO induced an ∼44% increase in cAMP-mediated Cl^−^ secretion in CF_residual_ tissues, which expressed at least one mild *CFTR* mutation producing Cl^−^ channels with residual function (Table 1, Fig. 4, 5). In contrast, consistent with findings in the colon of Cftr-deficient (Cftr^-/-^) mice [33], [34], 1-EBIO failed to induce Cl^−^ secretion in CF_absent_ tissues expressing two severe *CFTR* mutations that resulted in a lack of functional CFTR Cl^−^ channels in the luminal membrane of colonocytes (Table 1, Fig. 4, 5), including rectal tissues from patients homozygous for the common processing mutation F508del-CFTR. However, it is noteworthy that 1-EBIO was able to increase residual function of F508del-CFTR after low temperature correction in F508del-expressing human bronchial epithelial cells [45]. When viewed in combination, these results demonstrate that 1-EBIO, via activation of basolateral KCNN4 K^+^ channels, improves cAMP-mediated Cl^−^ secretion in human CF tissues expressing several CFTR mutants with residual Cl^−^ channel activity including F508del-CFTR when inserted into cell membrane.

The relevance of these findings is underscored by recent results driven from phase 2 clinical trials testing the CFTR potentiator VX-770 [12] and the CFTR corrector PTC124 [15] in CF patients carrying *CFTR* mutations responsive to these drugs. In these studies, measurements of nasal potential difference indicated that on average, both therapeutic strategies produced similar levels of functional correction of CFTR-mediated Cl^−^ secretion in the range of ∼20% of normal [12], [13], [15]. The results from previous studies correlating CF disease severity with CFTR-mediated Cl^−^ secretion in nasal and rectal epithelia predict that functional correction to this level (i.e. ∼20%) may ameliorate disease severity, but will be insufficient to prevent CF organ disease [16], [17]. Our data provide a proof-of-concept that pharmacological co-activation of basolateral KCNN4 K^+^ channels substantially potentiates residual CFTR function in native CF tissues (Fig. 4,5), and may thus provide an opportunity to improve therapeutic effects of current CFTR potentiator and corrector compounds developed either to improve the open probability (P_O_) or to increase the number of mutant CFTR Cl^−^ channels in the apical cell membrane [10], [46], [47]. However, no potent KCNN4 K^+^ channel openers have so far become available for clinical testing. While previous structure-activity studies were able to identify benzimidazolone derivatives that were up to 100-fold more potent in activating KCNN4 than the reference compound 1-EBIO (EC_50_ ∼600 µmol/l), these improvements were not sufficient for clinical development [48]. We expect that recent developments in functional high-throughput screening including cell-based fluorescent screens for K^+^ channel activity will enable the identification of new KCNN4 K^+^ channel activators that may be useful as lead compounds for CF drug development [46], [47], [49].

We previously demonstrated that transepithelial bioelectric measurements in rectal biopsies provide a sensitive tool to detect residual CFTR function in native tissues from individual CF patients carrying mild *CFTR* mutations, and that the magnitude of residual residual CFTR function correlated with clinical outcomes including age at diagnosis, exocrine pancreatic function, and lung function [17]. In these prior studies, the magnitute of normal cAMP-induced CFTR-mediated Cl^−^ secretion detected with our modified micro-Ussing chambers in rectal tissues from healthy individuals was ∼5 to 40-fold greater than values reported with other experimental setups [17], [36], [50], [51] indicating that our protocols detected CFTR function at high fidelity. However, the sensitivity of this assay to detect effects of CFTR potentiators and correctors was not known. Our present results indicate that this technique provides a sensitive tool to detect potentiator effects on low levels of residual CFTR function in individual CF patients (Fig. 4,5). These data suggest that assessment of CFTR function in rectal biopsies may provide a sensitive biomarker to evaluate effects of CFTR potentiator and corrector drugs in future clinical trials aimed at correcting the CFTR-associated defects [52]. We expect that this new outcome measure, together with bioelectric measurments of CFTR function in sweat glands and nasal epithelia [13], [53], will help to determine the level of functional correction that is required in different organ systems to achieve therapeutic benefits in CF patients. In the future, assessment of CFTR function in rectal biopsies and pre-testing of drugs *ex vivo* may also aid individualized CF therapy. In this context, this measurement could help to stratify CF patients carrying *CFTR* mutations with unknown functional consequences for treatment with CFTR potentiator or corrector drugs, and also pre-assess which patients will be ‘responders’ to a given drug by determining its therapeutic effects on CFTR function directly in native tissues from each individual CF patient.

In summary, we demonstrate that 1-EBIO activates CFTR-mediated Cl^−^ secretion in native human colon by coordinate activation of luminal CFTR Cl^−^ channels and basolateral Ca^2+^-activated KCNN4 K^+^ channels, and that this mode of action potentiates residual cAMP-mediated Cl^−^ secretion in native CF rectal tissues expressing CFTR mutants that retain residual Cl^−^ channel function. Our results suggest that pharmacological co-activation of KCNN4 K^+^ channels may augment therapeutic effects of CFTR potentiator and corrector compounds that are currently in active preclinical and clinical development, and suggest KCNN4 as a therapeutic target for CF.

## References

[1] Kerem B, Rommens JM, Buchanan JA, Markiewicz D, Cox TK (1989). Identification of the cystic fibrosis gene: genetic analysis.. Science.

[2] Welsh MJ, Ramsey BW, Accurso F, Cutting GR, Scriver CR, Beaudet AL, Sly WS, Valle D (2001). Cystic fibrosis.. The Metabolic & Molecular Bases of Inherited Disease. 8th ed.

[3] Riordan JR (2008). CFTR function and prospects for therapy.. Annu Rev Biochem.

[4] Cheng SH, Gregory RJ, Marshall J, Paul S, Souza DW (1990). Defective intracellular transport and processing of CFTR is the molecular basis of most cystic fibrosis.. Cell.

[5] Hamosh A, Trapnell BC, Zeitlin PL, Montrose-Rafizadeh C, Rosenstein BJ (1991). Severe deficiency of cystic fibrosis transmembrane conductance regulator messenger RNA carrying nonsense mutations R553X and W1316X in respiratory epithelial cells of patients with cystic fibrosis.. J Clin Invest.

[6] Sheppard DN, Rich DP, Ostedgaard LS, Gregory RJ, Smith AE (1993). Mutations in CFTR associated with mild-disease-form Cl^−^ channels with altered pore properties.. Nature.

[7] Welsh MJ, Smith AE (1993). Molecular mechanisms of CFTR chloride channel dysfunction in cystic fibrosis.. Cell.

[8] Kunzelmann K, Mall M (2002). Electrolyte transport in the mammalian colon: mechanisms and implications for disease.. Physiol Rev.

[9] Boucher RC (2007). Airway surface dehydration in cystic fibrosis: pathogenesis and therapy.. Annu Rev Med.

[10] Sloane PA, Rowe SM (2010). Cystic fibrosis transmembrane conductance regulator protein repair as a therapeutic strategy in cystic fibrosis.. Curr Opin Pulm Med.

[11] Van Goor F, Hadida S, Grootenhuis PD, Burton B, Cao D (2009). Rescue of CF airway epithelial cell function in vitro by a CFTR potentiator, VX-770.. Proc Natl Acad Sci U S A.

[12] Accurso FJ, Rowe SM, Clancy JP, Boyle MP, Dunitz JM (2010). Effect of VX-770 in persons with cystic fibrosis and the G551D-CFTR mutation.. N Engl J Med.

[13] Knowles MR, Paradiso AM, Boucher RC (1995). In vivo nasal potential difference: techniques and protocols for assessing efficacy of gene transfer in cystic fibrosis.. Hum Gene Ther.

[14] Du M, Liu X, Welch EM, Hirawat S, Peltz SW (2008). PTC124 is an orally bioavailable compound that promotes suppression of the human CFTR-G542X nonsense allele in a CF mouse model.. Proc Natl Acad Sci U S A.

[15] Kerem E, Hirawat S, Armoni S, Yaakov Y, Shoseyov D (2008). Effectiveness of PTC124 treatment of cystic fibrosis caused by nonsense mutations: a prospective phase II trial.. Lancet.

[16] Noone PG, Zhou Z, Silverman LM, Jowell PS, Knowles MR (2001). Cystic fibrosis gene mutations and pancreatitis risk: relation to epithelial ion transport and trypsin inhibitor gene mutations.. Gastroenterology.

[17] Hirtz S, Gonska T, Seydewitz HH, Thomas J, Greiner P (2004). CFTR Cl- channel function in native human colon correlates with the genotype and phenotype in cystic fibrosis.. Gastroenterology.

[18] MacVinish LJ, Hickman ME, Mufti DA, Durrington HJ, Cuthbert AW (1998). Importance of basolateral K+ conductance in maintaining Cl- secretion in murine nasal and colonic epithelia.. J Physiol.

[19] Mall M, Bleich M, Greger R, Schürlein M, Kühr J (1998). Cholinergic ion secretion in human colon requires co-activation by cAMP.. Am J Physiol.

[20] Devor DC, Singh AK, Gerlach AC, Frizzell RA, Bridges RJ (1997). Inhibition of intestinal Cl- secretion by clotrimazole: direct effect on basolateral membrane K+ channels.. Am J Physiol.

[21] Joiner WJ, Wang LY, Tang MD, Kaczmarek LK (1997). hSK4, a member of a novel subfamily of calcium-activated potassium channels.. Proc Natl Acad Sci.

[22] Warth R, Hamm K, Bleich M, Kunzelmann K, von Hahn T (1999). Molecular and functional characterization of the small Ca(2+)-regulated K+ channel (rSK4) of colonic crypts.. Pflügers Arch.

[23] Flores CA, Melvin JE, Figueroa CD, Sepulveda FV (2007). Abolition of Ca2+-mediated intestinal anion secretion and increased stool dehydration in mice lacking the intermediate conductance Ca2+-dependent K+ channel Kcnn4.. J Physiol.

[24] Matos JE, Sausbier M, Beranek G, Sausbier U, Ruth P (2007). Role of cholinergic-activated KCa1.1 (BK), KCa3.1 (SK4) and KV7.1 (KCNQ1) channels in mouse colonic Cl- secretion.. Acta Physiol (Oxf).

[25] Lohrmann E, Burhoff I, Nitschke RB, Lang H-J, Mania D (1995). A new class of inhibitors of cAMP-mediated Cl^−^ secretion in rabbit colon, acting by the reduction of cAMP-activated K^+^ conductance.. Pflugers Arch.

[26] Bleich M, Briel M, Busch AE, Lang H-J, Gerlach U (1997). K_V_LQT channels are inhibited by the K+ channel blocker 293B.. Pflügers Arch.

[27] Schroeder BC, Waldegger S, Fehr S, Bleich M, Warth R (2000). A constitutively open potassium channel formed by KCNQ1 and KCNE3.. Nature.

[28] Vallon V, Grahammer F, Volkl H, Sandu CD, Richter K (2005). KCNQ1-dependent transport in renal and gastrointestinal epithelia.. Proc Natl Acad Sci U S A.

[29] Preston P, Wartosch L, Gunzel D, Fromm M, Kongsuphol P (2010). Disruption of the K+ channel beta-subunit KCNE3 reveals an important role in intestinal and tracheal Cl- transport.. J Biol Chem.

[30] Heitzmann D, Warth R (2008). Physiology and pathophysiology of potassium channels in gastrointestinal epithelia.. Physiol Rev.

[31] Devor DC, Singh AK, Frizzell RA, Bridges RJ (1996). Modulation of Cl^−^ secretion by benzimidazolones. I. Direct activation of a Ca^2+^- dependent K^+^ channel.. Am J Physiol.

[32] Devor DC, Singh AK, Bridges RJ, Frizzell RA (1996). Modulation of Cl- secretion by benzimidazolones. II. Coordinate regulation of apical GCl and basolateral GK.. Am J Physiol.

[33] Cuthbert AW, Hickman ME, Thorn P, MacVinish LJ (1999). Activation of Ca(2+)- and cAMP-sensitive K(+) channels in murine colonic epithelia by 1-ethyl-2-benzimidazolone.. Am J Physiol.

[34] MacVinish LJ, Keogh J, Cuthbert AW (2001). EBIO, an agent causing maintained epithelial chloride secretion by co-ordinate actions at both apical and basolateral membranes.. Pflugers Arch.

[35] Cuthbert AW (2001). Assessment of CFTR chloride channel openers in intact normal and cystic fibrosis murine epithelia.. Br J Pharmacol.

[36] Mall M, Kreda SM, Mengos A, Jensen TJ, Hirtz S (2004). The DeltaF508 mutation results in loss of CFTR function and mature protein in native human colon.. Gastroenterology.

[37] Rosenstein BJ, Cutting GR (1998). The diagnosis of cystic fibrosis: a consensus statement.. J Pediatr.

[38] De Boeck K, Wilschanski M, Castellani C, Taylor C, Cuppens H (2006). Cystic fibrosis: terminology and diagnostic algorithms.. Thorax.

[39] Wallis C, Leung T, Cubitt D, Reynolds A (1997). Stool elastase as a diagnostic test for pancreatic function in children with cystic fibrosis.. Lancet.

[40] Mall M, Wissner A, Seydewitz HH, Kuehr J, Brandis M (2000). Defective cholinergic Cl^−^ secretion and detection of K^+^ secretion in rectal biopsies from cystic fibrosis patients.. Am J Physiol Gastrointest Liver Physiol.

[41] Mall M, Wissner A, Seydewitz HH, Hübner M, Kuehr J (2000). Effect of genistein on native epithelial tissue from normal individuals and CF patients and on ion channels expressed in Xenopus oocytes.. Br J Pharmacol.

[42] Mall M, Gonska T, Thomas J, Schreiber R, Seydewitz HH (2003). Modulation of Ca^2+^-activated Cl^−^ secretion by basolateral K^+^ channels in human normal and cystic fibrosis airway epithelia.. Pediatr Res.

[43] Mall M, Wissner A, Schreiber R, Kuehr J, Seydewitz HH (2000). Role of K_V_LQT1 in cAMP-mediated Cl^−^ secretion in human airway epithelia.. Am J Respir Cell Molec Biol.

[44] von Hahn T, Thiele I, Zingaro L, Hamm K, Garcia-Alzamora M (2001). Characterisation of the rat SK4/IK1 K(+) channel.. Cell Physiol Biochem.

[45] Devor DC, Bridges RJ, Pilewski JM (2000). Pharmacological modulation of ion transport across wild-type and ΔF508 CFTR-expressing human bronchial epithelia.. Am J Physiol Cell Physiol.

[46] Becq F (2006). On the discovery and development of CFTR chloride channel activators.. Curr Pharm Des.

[47] Verkman AS, Lukacs GL, Galietta LJ (2006). CFTR chloride channel drug discovery--inhibitors as antidiarrheals and activators for therapy of cystic fibrosis.. Curr Pharm Des.

[48] Singh S, Syme CA, Singh AK, Devor DC, Bridges RJ (2001). Benzimidazolone activators of chloride secretion: potential therapeutics for cystic fibrosis and chronic obstructive pulmonary disease.. J Pharmacol Exp Ther.

[49] Namkung W, Padmawar P, Mills AD, Verkman AS (2008). Cell-based fluorescence screen for K+ channels and transporters using an extracellular triazacryptand-based K+ sensor.. J Am Chem Soc.

[50] Veeze HJ, Sinaasappel M, Bijman J, Bouquet J, De Jonge HR (1991). Ion transport abnormalities in rectal suction biopsies from childern with cystic fibrosis.. Gastroenterology.

[51] Derichs N, Sanz J, Von Kanel T, Stolpe C, Zapf A (2010). Intestinal current measurement for diagnostic classification of patients with questionable cystic fibrosis: validation and reference data.. Thorax.

[52] Amaral MD (2011). Targeting CFTR: How to Treat Cystic Fibrosis by CFTR-Repairing Therapies.. Curr Drug Targets.

[53] Gonska T, Ip W, Turner D, Han WS, Rose J (2009). Sweat gland bioelectrics differ in cystic fibrosis: a new concept for potential diagnosis and assessment of CFTR function in cystic fibrosis.. Thorax.

